# Glass Interposer Assisted Edge Coupling to SiN Photonic Integrated Circuits

**DOI:** 10.1021/acsphotonics.5c02702

**Published:** 2026-03-27

**Authors:** Ipsita Chakraborty, Elliot Sandell, Thalía Domínguez Bucio, Glenn Churchill, Xingshi Yu, Michail Symeonidis, Hawraa Atwi, Clement Fresse, James Gates, Frederic Gardes

**Affiliations:** † Optoelectronics Research Centre, 7423 University of Southampton, Southampton SO17 1BJ, United Kingdom; ‡ 28424 Fraunhofer Institute for Reliability and Microintegration IZM, Gustav-Meyer-Allee 25, Berlin 13355, Germany; § TEEM Photonics, 61 Chemin du Vieux Chêne, Meylan Cedex 38240, France

**Keywords:** fiber-to-chip coupling, edge couplers, silicon nitride (SiN) photonics, waveguide array to fiber (WAFT) interposer, high-density photonic interconnects, scalable photonic packaging

## Abstract

Scaling the fiber-to-chip interface is a critical bottleneck for modern photonic interconnect solutions, which must support the growing density and bandwidth demands of high-performance photonic integrated circuit (PIC) applications. In this study, we present a novel, high-density edge coupling solution that enables low-loss, simultaneous multichannel coupling to a silicon nitride (SiN) chip, utilizing a glass-based waveguide array to fiber (WAFT) interposer. The WAFT interposer efficiently bridges single-mode fibers to the SiN chip, providing mode-field matching and supporting an exceptional density of up to 67 input/output (I/O) channels per millimeter for robust, scalable integration. We designed and fabricated SiN edge couplers optimized for the WAFT-coupled fibers and characterized the full coupling interface—from fiber through the WAFT to the SiN chip across the O and C optical bands. Measured fiber-to-chip coupling loss (CL) per optical port ranged from 1.55–1.81 dB (TE)/1.55–1.93 dB (TM) in the O band and 1.67–1.86 dB (TE)/1.58–1.69 dB (TM) in the C band. Furthermore, polarization-dependent loss (PDL) tests showed low CL of 1.42–1.62 dB/optical port at 1310 nm and 1.35–1.83 dB/optical port at 1550 nm. These results indicate that the WAFT-based edge coupling solution holds significant promise as an effective, scalable, and bandwidth-flexible approach, potentially addressing a critical packaging bottleneck for advanced, high-density PIC integration.

## Graphical abstract

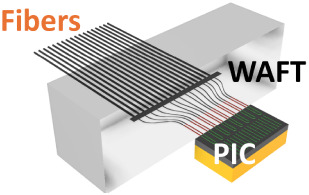

## 1 Introduction

In recent years, photonic integrated circuits (PICs) have evolved to support a broad range of data- and bandwidth-intensive applications, ranging from data centers and high-speed computing to sensing and quantum technologies. Leveraging submicron fabrication techniques, PICs can integrate hundreds of optical components within compact footprints, achieving unprecedented levels of on-chip functionality. However, as PICs scale toward higher integration densities, the limitations of conventional fiber interconnects become increasingly pronounced in terms of port scalability.^1,2^ Single-mode fibers (SMF) have cladding diameter of 125 μm or more which makes them incompatible with the fine-pitch requirements of densely packed PIC interfaces. An illustrative example is that a next-generation 51.2 Tbps switch ASIC may assemble 16 3.2 Tbps PICs, each requiring 16 fibers (eight for 400 Gbps transmitters and eight for receivers), for a total of 256 fibers to manage at the front panel.^3,4^ This scenario underscores the urgency for scalable, efficient, and compact photonic interconnects that can accommodate large optical fibers within compact photonic chip interfaces, supporting the future of high-bandwidth communication systems.

The two widely employed fiber to PIC coupling approaches are surface coupling (e.g., grating or evanescent methods) and edge coupling. Grating couplers (GCs) offer relaxed fiber alignment and support wafer-scale testing but they suffer from limited bandwidth and polarization sensitivity, particularly in 1D implementations. Subwavelength engineered 1D-GCs can reduce polarization dependence, while 2D grating couplers^5^ offer a robust alternative when polarization is unknown or unstable. Evanescent couplers also deliver low coupling loss (CL) of 0.8 dB/optical port across the C and L bands,^6^ making them attractive for broadband applications. However, their large footprint limits their suitability for ultradense PIC integration.

In contrast, edge couplers offer high coupling efficiency, broadband operation and polarization independence.^7^ PIC integrates spot size converters (SSCs) that expand mode field diameters at the chip edge from ∼2–5 to 10 μm to match the single-mode fibers (SMFs).^8–15^ Several edge mode size converters have been reported, including inverse tapers combined with specialty fibers such as lensed or ultrahigh numerical aperture (UHNA) fibers, which help to minimize CL. Dual-tip tapers with UHNA fiber gave 1.9 dB/optical port loss across O band.^16^ Other designs, such as hybrid edge couplers with subwavelength gratings,^17^ ribbed SiN/silica claddings interfaced with high numerical aperture (NA) fiber,^18^ and 3D prong-like converters^19^ further minimize CL to below 1 dB.

Etched V-grooves in PIC substrates^20,21^ provide a cost-effective, passive solution for fiber-to-chip alignment, particularly in high-volume manufacturing. They simplify packaging and enable stable fiber placement, but their relatively large footprint can limit scalability in high-density PIC applications that require compact optical port layouts. Reported theoretical CL for these structures are 3.5 dB (TE) and 3.7 dB (TM).^20^ Two-photon polymerization (2PP)^22^ offers a complementary approach by enabling the fabrication of 3D tapers directly on SMF facets, achieving CL of 1.65 dB (linear taper) and 2.09 dB (with lensed fiber). However, their mechanical fragility remains a limitation for robust packaging.

A compact alternative mode field matching solution between the SiN PIC and fibers is using glass-based waveguide array to fiber interposers (WAFT), developed by TEEM Photonics.^23^ WAFTs integrate spot-size converters and fiber-spacing concentrators, allowing customizable channel pitch and port count without modifying the external fiber array. They enable a gradual mode field diameter (MFD) transition from 10 μm (fiber side) to smaller values at the PIC interface (see Table 1), supporting pitches as small as 15 μm and up to 121 channels, ideal for high-density optical I/O in a compact footprint. Table S3 of the Supporting Information (SI) presents the parameters of the internal WAFT section, which consists of a buried ion-diffused waveguide.

**Table 1:** Effective Channel Width, Effective Refractive Index, and MFD of the WAFT Channel Waveguide at the PIC Interface

Configuration	WAFT Channel Width (μm)	Effective Index	Horizontal MFD (μm)	Vertical MFD (μm)
TE 1310 nm	2	1.525025	3.79	2.73
TM 1310 nm	2	1.524825	3.75	2.80
TE 1550 nm	2	1.518483	4.29	3.13
TM 1550 nm	2	1.518254	4.25	3.23

We present the design and fabrication of SiN edge couplers optimized for WAFT integration. Experimental characterization of the full optical path from input fiber through WAFT to the SiN PIC was performed across the O and C bands for both TE and TM polarization. CL, broadband performance, and polarization-dependent loss (PDL) were evaluated to demonstrate the performance of our edge coupler approach for polarization-diverse and scalable fiber-to-chip integration enabled by WAFT compatibility.

## 2 Experimental Section

Figure 1a shows the three-dimensional schematic of the EC configuration, illustrating the glass-based WAFT bridging single-mode fibers to the SiN photonic chip. The WAFT incorporates a buried ion-diffused channel waveguide having a width of 2 μm. The channel width, effective refractive index, and MFD of the WAFT channel waveguide are summarized in Table 1. The photonic chip features an oxide-cladded SiN waveguide of thickness 400 nm (H). A 2 μm oxide cladding ensures effective confinement of the optical mode within the SiN waveguide. Thicker oxide claddings were investigated using Ansys Lumerical eigenmode Expansion (EME) simulations (see Figure S6 of Supporting Information) and were found to provide only marginal improvement in coupling efficiency. Increasing the cladding thickness also leads to higher film stress and greater fabrication cost, therefore, a 2 μm oxide cladding was selected as an optimal trade-off. The entire structure rests on a 3.2 μm oxide BOX and silicon substrate. SiN inverse tapers were designed to facilitate efficient edge coupling between the photonic chip and the WAFT. The SiN waveguide width was gradually tapered from W = 700 nm (O band) and W = 1000 nm (C band) to a tip width “t”, over a length L, which was optimized using the Ansys Lumerical EME solver. The taper tip width “t” and the relative position of the WAFT channel with respect to the SiN taper were optimized using a Finite Difference eigenmode (FDE) solver by maximizing the modal overlap between the SiN waveguide and the WAFT channel waveguide. At 1310 nm wavelength, t was varied from 210 to 310 nm in 20 nm increments for both TE and TM polarizations. As shown in Figure 1b, for 1310 nm wavelength, the mode overlap for both TE and TM polarization decreases with increasing t, indicating that smaller taper tip widths are favorable. However, due to the critical dimension limit of the deep-UV lithography scanner (approximately 200 nm), the minimum taper tip width investigated at 1310 nm wavelength was limited to 210 nm. As width smaller than 200 nm would compromise the structural integrity and fabrication yield. At 1550 nm wavelength, t was varied from 250 to 350 nm in 20 nm increments. The TE mode overlap initially increases with increasing t, reaching a maximum at t = 290 nm, before decreasing for larger values. In contrast, the TM mode overlap decreases monotonically with increasing t at 1550 nm wavelength. Next, the taper length L was optimized using the Lumerical eigenmode Expansion (EME) solver. For each value of t considered, L was swept from 0 to 500 μm in 1 μm steps, and the corresponding transmission was calculated. Figure 1b shows the transmission as a function of taper length for both TE and TM polarization at t = 230 at 1310 nm and t = 290 at 1550 nm. The results indicate that transmission increases with L and stabilizes for lengths exceeding 200 μm in all four cases. Then, we performed 3D FDTD simulations to evaluate the CL of the proposed EC configuration between a WAFT and an oxide-cladded SiN taper. For both TE and TM polarizations, L was fixed at 200 μm, with t = 230 nm for 1310 nm and t = 290 nm for 1550 nm. The resulting CL values were 0.613 dB (TE)/1.349 dB (TM) at 1310 nm and 0.59 dB (TE)/0.97 dB (TM) at 1550 nm. Experimentally, to measure the fiber-to-chip CL, we employed a cut-back method incorporating two SSCs, each comprising an inverse tapered waveguide. These SSCs were placed in parallel with a spacing of 127 μm, matching the pitch of the WAFT channel waveguides. The tapers were positioned adjacent to a straight waveguide and connected via a bend with a radius of 63.5 μm. A 40 μm straight waveguide was added at each taper facet to ensure smooth mode transition from the WAFt to the SiN taper. To quantify bend loss (BL) and taper loss (TL) at 1310 and 1550 nm, a suite of test structures was designed in our mask layout for both TE and TM polarization, as shown in Figure 2a–c. For bend loss evaluation, the number of bends was varied from N = 1 to N = 10. To assess the optical loss introduced by the taper section and to measure how efficiently light transitions through the tapered region, we included an array of back-to-back taper structures. For each variation in t and L, the number of connected back-to-back tapers was systematically increased in multiples of two, up to a maximum of N = 16, resulting in 32 individual taper transitions. The measured PL, BL and TL are summarized in Table 2 along with their associated standard error, at both 1310 and 1550 nm.

**Table 2:** Measured PL, BL, and TL Values at 1550 and 1310 Nm

Configuration	PL (dB/cm)	BL (dB/bend)	TL^a^ (dB/taper)
TE 1310 nm	0.44 ± 0.03	0.07 ± 0.05	0.23 ± 0.04
TM 1310 nm	0.59 ± 0.02	0.09 ± 0.06	0.25 ± 0.06
TE 1550 nm	0.35 ± 0.05	0.05 ± 0.06	0.20 ± 0.03
TM 1550 nm	0.35 ± 0.02	0.09 ± 0.08	0.14 ± 0.09

a TL measured for t = 230 nm (at 1310 nm) and t = 290 nm (at 1550 nm).

**Figure 1:**
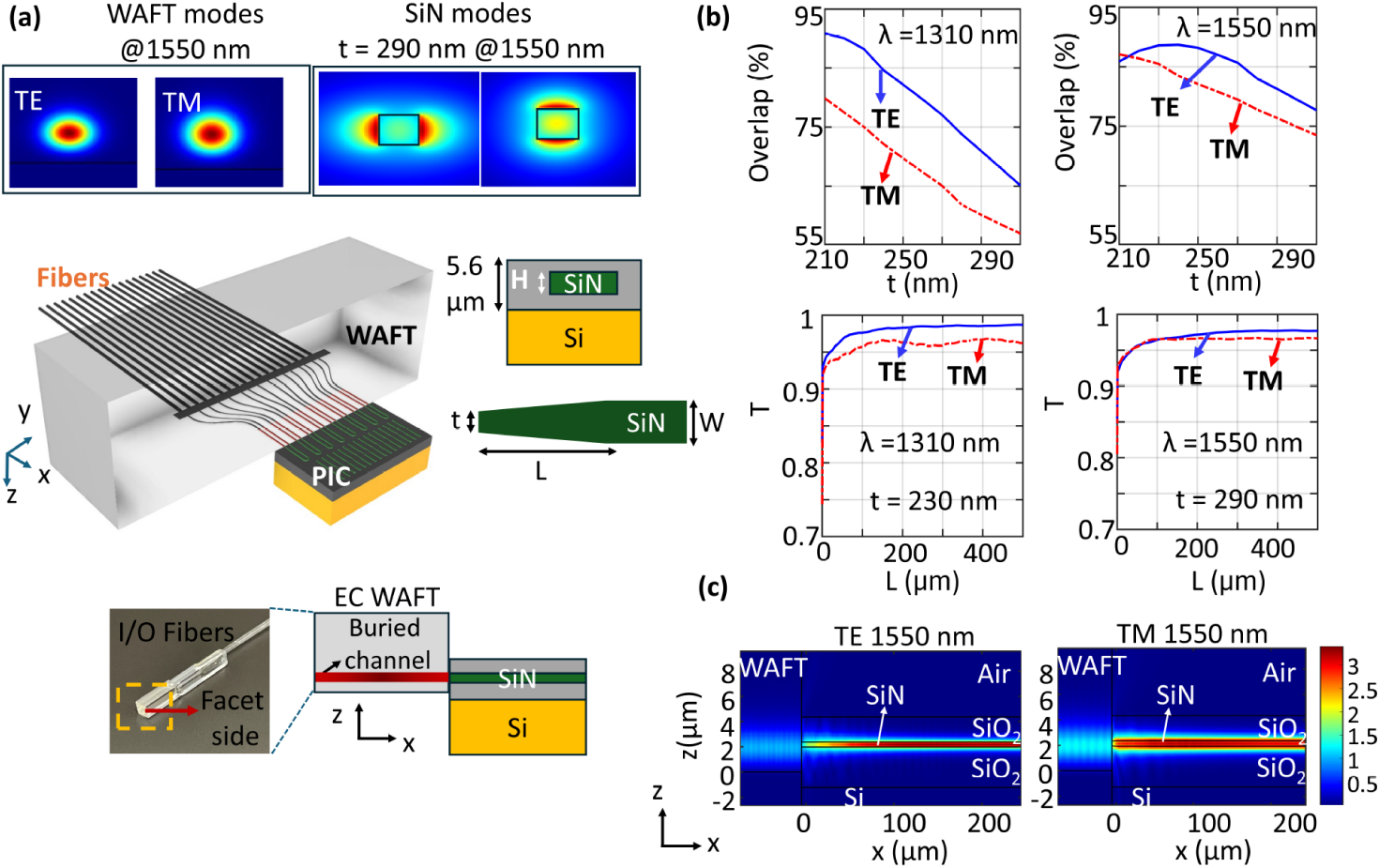
(a) A 3D model of the edge coupler configuration is shown, illustrating the coupling between the buried channel waveguide in WAFT and the SiN waveguide. The top left inset highlights the WAFT modes at the PIC edge and the SiN modes for t = 290 nm, for both TE and TM polarizations at an operating wavelength of 1550 nm. The bottom left image shows TEEM manufactured WAFT having buried channel waveguides as well as input and output (I/O) optical fibers. The bottom-right image shows a 2D schematic of light coupling between the WAFT and the SiN chip, where x indicates the light propagation direction and z represents the vertical height. (b) Simulated mode overlap (%) between the WAFT mode and the TE/TM modes of a SiN taper tip of width “t” using Ansys Lumerical FDE. Effect of taper length on transmission for TE and TM modes, simulated using the Lumerical eigenmode expansion solver, at 1310 nm wavelength with t = 230 nm and at 1550 nm wavelength with t = 290 nm. (c) Simulated electric field propagation of TE and TM modes in EME through a SiN taper and WAFT structure, with L = 200 μm and t = 290 nm at 1550 nm.

**Figure 2:**
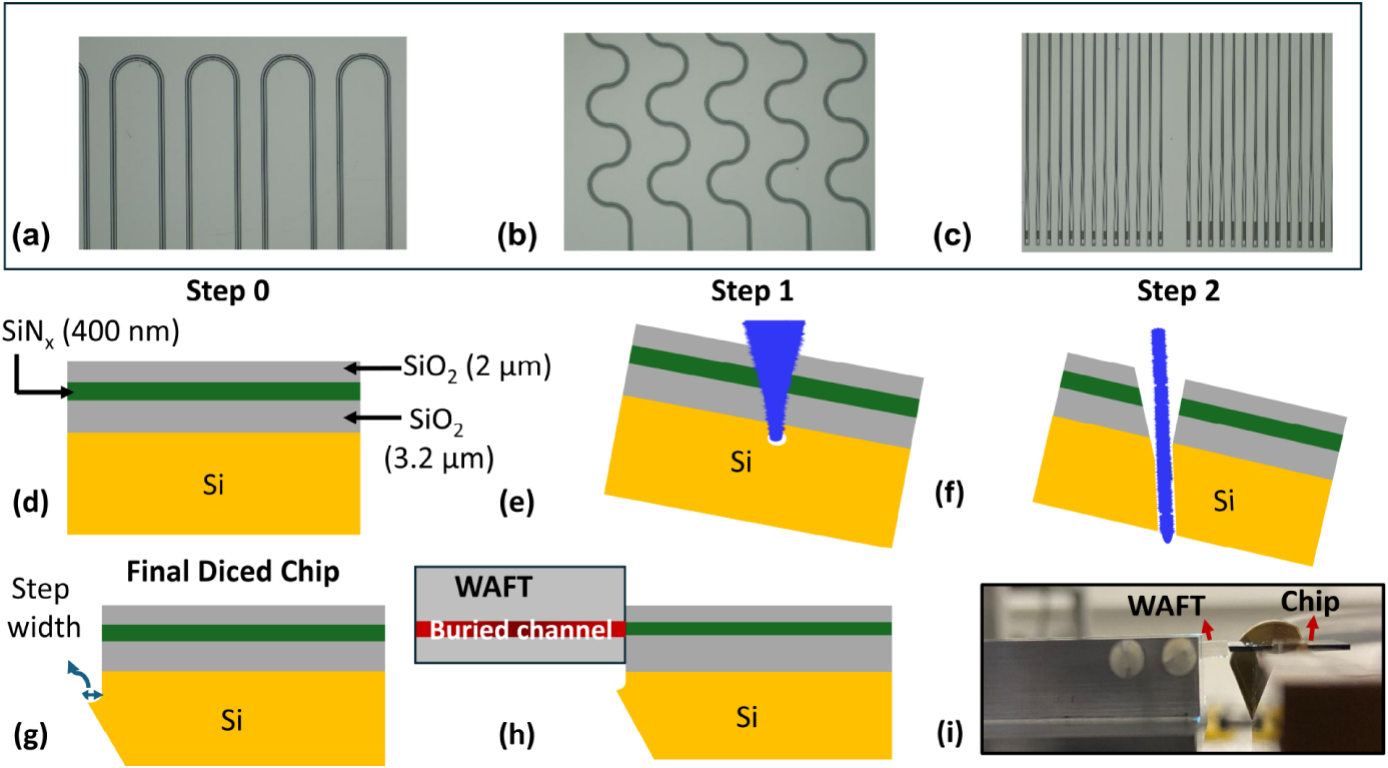
(a–c) Microscopic images of a SiN photonic chip featuring test structures designed to assess (a) taper width loss, (b) bend loss, and (c) taper loss. Sequential dicing steps for achieving a high quality end facet cut on a silicon nitride photonic chip: (d) Step 0 indicates the layer stack and thickness of the SiN-based chip. (e) The chip is mounted at an angle, and a 16 μm deep ductile dicing cut is made. Imperfect blade form results in a cut normal to the top surface, marked as Step 1. (f) Next, in Step 2, a full depth singulation cut using a thin blade is performed within the boundaries of the initial cut to isolate the chip. (g) A slight step of width up to 4 μm may remain as shown in the final diced chip, due to machine inaccuracies. (h) The WAFT is flipped upside down to ensure alignment between the buried channel waveguide of the WAFT and the oxide cladded silicon nitride waveguide (i) Final EC setup between WAFT and our SiN photonic chip.

Several techniques are available for EC facet preparation, including conventional polishing and ductile dicing. For our devices, ductile dicing^24^ was selected due to its ability to produce high-quality facets with high precision and minimal top surface chipping. The step-by-step process for facet preparation and die singulation is illustrated in Figure 2d–f, with final diced chip shown in Figure 2g. The WAFT is then flipped upside down and placed at the chip edge so that its buried waveguide channels align with the SiN tapered waveguides as shown in Figure 2h. Fine registration is then performed through visual alignment of the chip edges and monitoring of the optical coupling efficiency. The final EC setup between WAFT and our SiN photonic chip is shown in Figure 2i. Following device fabrication, we then proceeded to characterize the EC performance.

## 3 Results and Discussion

Figure 3a shows the experimental setup used to evaluate the EC performance between SiN-based waveguide tapers and the WAFT interfaced with polarization-maintaining (PM) fibers at 1310 and 1550 nm wavelength. The measured insertion loss (IL) of the WAFT channel waveguide, including the PM fiber interfaces, is 0.95 dB at 1550 nm. Linearly polarized light from a tunable laser is delivered through PM fibers to a fiber bench equipped with a half-wave plate (HWP) and a polarization beam splitter (PBS) cube. The PBS is positioned on the fiber bench, with its dotted face facing upward. In this configuration, light entering the PBS along the horizontal axis is split based on polarization: TE-polarized light (p-polarized, with the electric field parallel to the chip plane) is transmitted straight through, while TM-polarized light (s-polarized, with the electric field perpendicular to the chip plane) is reflected at 90°, exiting through the side face in the same horizontal plane. The polarized output from the fiber bench is routed to a detector via PM fibers to monitor the transmitted power. By adjusting the HWP angle, the polarization state can be tuned to selectively launch either TE or

**Figure 3:**
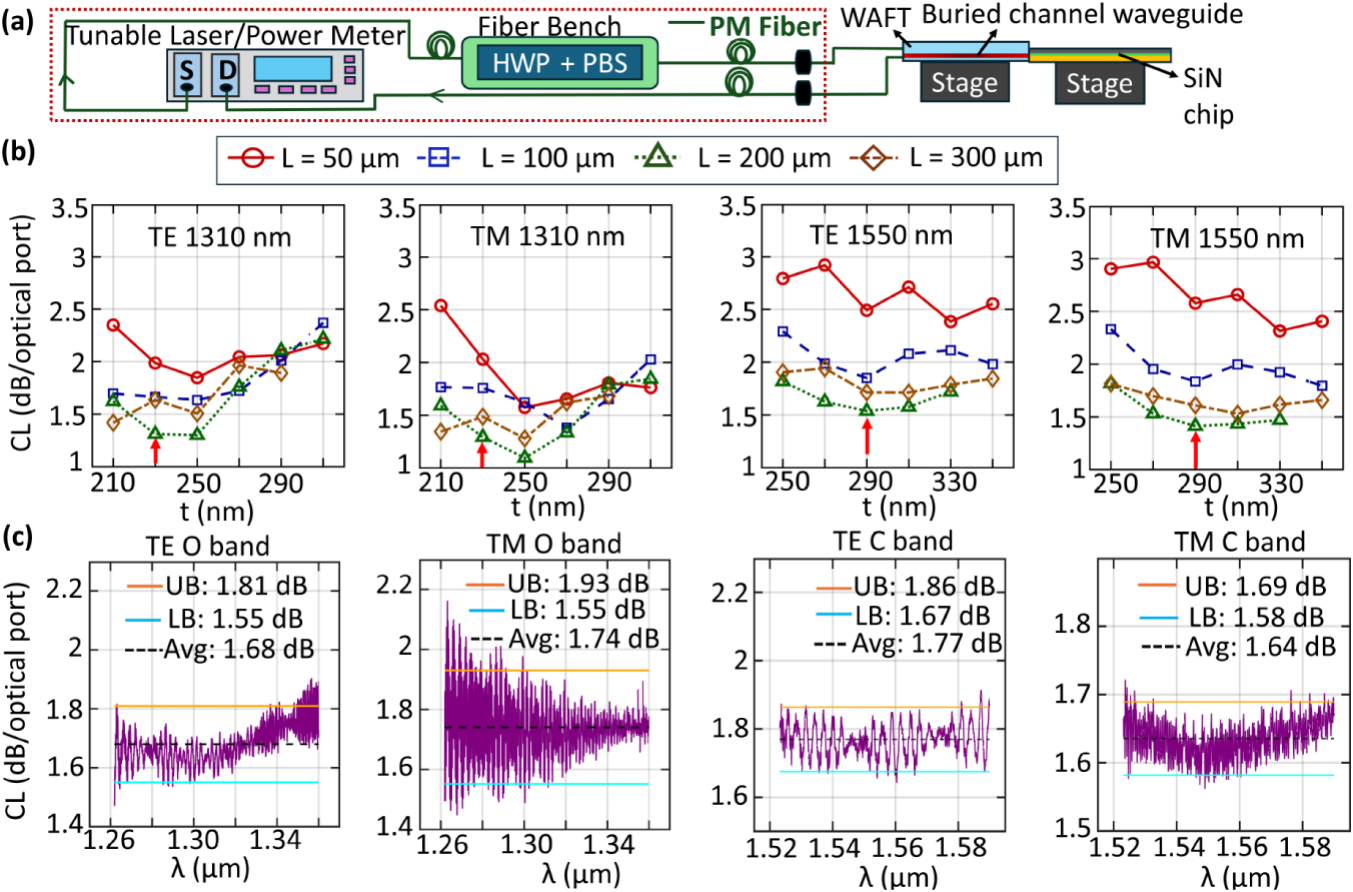
(a) Experimental setup used for CL measurements. Optical components are interconnected via PMF fibers, denoted by green lines. The dashed red box indicates the optical path included in the normalization loss calculation. This normalization accounts for the losses introduced by the tunable laser and power meter, fiber connectors and mating interfaces, polarization-maintaining fibers, and the fiber bench components including the half waveplate and polarization beam splitter. It also includes losses due to finite coupling efficiency during free space propagation, as well as losses from the fiber-port collimator and laser-port connectors. S and D denote source and detector in tunable laser and power meter. (b) CL dependence on t for various L at 1310 and 1550 nm wavelengths for both TE and TM polarizations. Red arrow indicates the optimal point for each case. (c) Broadband coupling loss evaluation across the O band and C band for TE and TM polarizations. L = 200 μm for all cases and t = 290 nm for 1550 nm and t = 230 nm for 1310 nm.

TM modes. Once the HWP orientation is adjusted for both TE and TM modes, the fiber bench output is then connected to the input PM fiber of the WAFT, which is edge-coupled to the SiN photonic chip. The output PM fiber from the WAFT is then connected to a detector to measure the output coupled power using the cut-back method as discussed in previous section. The CL, representing the fiber-to-SiN chip CL in decibels per optical port (dB/optical port), is calculated using the following relations (eq 1):

$$\mathrm{CL}=\frac{\mathrm{Total Loss}-\mathrm{Normalization Loss}-2\cdot \mathrm{TL}-\mathrm{BL}-2\cdot \left(\mathrm{PL}\cdot L_{s}\cdot 10^{-4}\right)}{2}$$ 1

Here PL is expressed in dB/cm and L_s_ in μm is the length of the straight SiN waveguide between each taper and its adjacent bend. L_s_ is converted to cm by multiplication with 10^–4^. The PL of the short straight segments immediately in front of the taper tips, with widths <1 μm, is expected to be slightly higher than that of the wider (700 nm for O band and 1 μm for C band) waveguides, however their lengths are very short (≈40 μm) and thus their contribution to the total loss is neglected in eq 1. Thus, eq 1 isolates the fiber-to-chip coupling loss by subtracting the normalization loss, TL, BL and PL (detailed in Table 2) within the silicon nitride waveguide, thereby capturing only the cumulative losses at the fiber–WAFT and WAFT–chip interfaces. Uncertainties in PL, BL, and TL (Table 2) are propagated to calculate the total CL uncertainty, as given by eq 2:

2 $$\sigma_{\mathrm{CL}}=\sqrt{\frac{\sigma_{\mathrm{BL}}^{2}}{4}+\sigma_{\mathrm{TL}}^{2}+{\left(\sigma_{\mathrm{PL}}\cdot L_{s}\cdot 10^{-4}\right)}^{2}}$$

Figure 3b presents the dependence of CL on taper tip width t across varying taper lengths L at 1310 and 1550 nm wavelengths. At 1310 nm, optimal coupling performance was achieved at t = 230 nm and L = 200 μm, resulting in fiber-to-chip CL of 1.31 ± 0.05 dB (TE) and 1.29 ± 0.07 dB per optical port (TM). At 1550 nm, t = 290 nm and L = 200 μm yielded CL per optical port of 1.53 ± 0.04 dB (TE) and 1.41 ± 0.1 dB (TM). Figure 3c shows the measured broadband coupling loss (CL) across the O and C bands for both TE and TM polarizations. The taper length was fixed at L = 200 μm, with taper tip widths of t = 230 nm for the O band and 290 nm for the C band. As detailed in Figure S4 of Supporting Information, BL, TL, and PL exhibit measurable wavelength dependence across both bands due to changes in mode confinement, effective index, and taper adiabaticity away from the design wavelength −1310 and 1550 nm. The broadband CL results were obtained without subtracting BL, TL, or PL in eq 1. Thus, the broadband CL in Figure 3c inherently includes the wavelength-dependent contributions of these losses. This leads to a slightly higher CL and modest spectral variation, particularly near the band edges. The broadband CL measurements reflect the true operational behavior of the device over the full wavelength range of interest. In the O band, TE losses varied between 1.55 to 1.81 dB/optical port (avg. 1.68 dB), and TM losses ranged from 1.55 to 1.93 dB/optical port (avg. 1.74 dB). For the C band, TE polarization losses ranged from 1.67 to 1.86 dB/optical port (avg. 1.77 dB), while TM losses ranged from 1.58 to 1.69 dB/optical port (avg. 1.64 dB). We performed a fast Fourier transform (FFT) of the broadband coupling-loss spectra in Figure 3c across the O and C bands for both TE and TM polarizations to quantitatively analyze the observed spectral oscillations using a Fabry–Perot interference model (see Subsection S1a under Section S1 of the Supporting Information for detailed analysis). The analysis indicates that these oscillations arise primarily from air-gap-induced Fresnel reflections and internal reflections at transitions between waveguide components, such as taper-to-straight and straight-to-bend sections. These oscillations can be mitigated by applying an index-matching liquid or adhesive at the air–waveguide interface (see Subsection S1f under Section S1 of the Supporting Information for candidate materials and long-term reliability), optimizing waveguide transitions, and employing Euler bends instead of constant-radius bends to further suppress reflections within the SiN cavity.

To evaluate the polarization sensitivity of the proposed EC scheme, polarization-dependent loss (PDL) measurements were conducted using the setup shown in Figure 4a. This characterization quantifies CL under varying input polarization states (Figure 4b,c), which is essential for ensuring stable performance and seamless system-level integration. A separate WAFT structure interfaced with standard SMF was employed for this measurement, exhibiting an IL of 0.7 dB at 1550 nm. PDL measurements at 1310 and 1550 nm were performed by identifying the maximum, minimum, and differential IL across all polarization states, leveraging the autoscanning feature of the Agilent HP11896A Polarization State Controller in combination with the Agilent HP8509B Polarization Analyzer. At 1310 nm, for a taper length L = 200 μm, polarization scans showed that a tip width of 230 nm yielded minimum and maximum coupling losses of 1.42 and 1.62 dB per optical port, respectively. In contrast, the same taper length with t = 250 nm exhibited a broader loss range, from 1.02 to 1.97 dB per optical port. Similarly, at 1550 nm, t = 290 nm produced the lowest observed losses, with minimum and maximum values of 1.35 and 1.83 dB per optical port, respectively. During the PDL measurement, the reported CL value is slightly higher than in Setup 1 because it includes losses from bends, tapers, and waveguide propagation. The broadband PDL across the O and C bands is shown in Figure S3 of the Supporting Information. Overall, the PDL remains low and varies smoothly with wavelength, showing no monotonic increase or decrease across either band.

**Figure 4:**
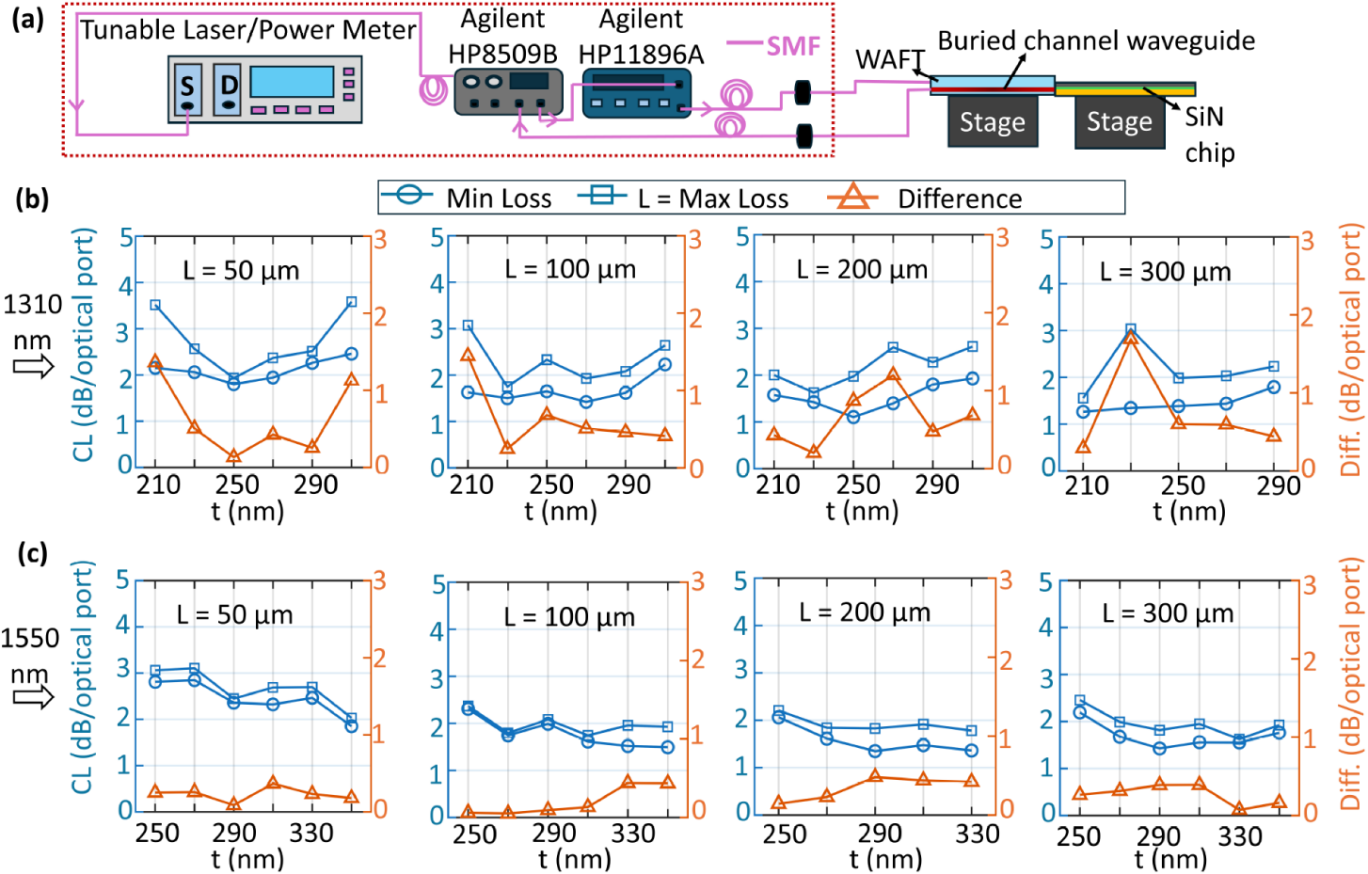
(a) Experimental setup for single-wavelength polarization-dependent loss (PDL) measurements at 1310 and 1550 nm. All optical components are connected using single-mode fibers (pink lines). The red dashed box denotes the combined normalization losses of the enclosed components, which are subtracted from the total coupling loss. These include losses from the tunable laser and power meter, fiber connectors and mating interfaces, single-mode fibers, the polarization controller (HP 11896A), and the polarization analyzer (HP 8509B). BL, TL, and PL are not subtracted from the reported CL because PDL measurements are obtained from a full polarization scan, whereas BL, TL, and PL were characterized only for TE and TM polarizations. (b–c) Maximum, minimum, and differential loss as a function of t for different values of L at 1310 and 1550 nm.

During both CL and PDL measurements, the EC-WAFT was secured on a tilt stage using mechanical screws, while the SiN chip was mounted on a separate vacuum stage. No adhesive was used between the WAFT and the SiN chip, so small variations in lateral position or tilt inevitably occurred during repeated alignments. To evaluate the sensitivity of CL and PDL to such alignment variations, we performed ten repeated measurements on the same WAFT–SiN chip pair, switching between different taper widths and reconnecting the fibers each time. These ten repeated measurements were conducted on both experimental setups, as shown in Figure 3a and Figure 4a, to assess measurement repeatability and to account for losses arising from procedural factors, such as repeated fiber disconnection and reconnection at the connectors and laser assemblies during individual measurements. The taper parameters were set to t = 230 nm, L = 200 μm for 1310 nm, and t = 290 nm, L = 200 μm for 1550 nm across both setups. The uncertainty of the total insertion loss, σ_IL_ was estimated as the standard error of the mean from ten repeated measurements on the same SiN chip and WAFT device, capturing variability due to fiber disconnection, realignment, and power-meter stability. The total uncertainty in CL is then calculated by combining σ_IL_ with the PL, BL, and TL uncertainties as shown in eq 3:

3 $$\sigma_{\mathrm{CL}}=\sqrt{\sigma_{\mathrm{IL}}^{2}+\frac{\sigma_{\mathrm{BL}}^{2}}{4}+\sigma_{\mathrm{TL}}^{2}+{\left(\sigma_{\mathrm{PL}}\cdot L_{s}\cdot 10^{-4}\right)}^{2}}$$

For setup 1 (Figure 3a), the measured mean CL at 1310 nm were 1.29 ± 0.06 dB/optical port (TE) and 1.22 ± 0.08 dB/optical port (TM). At 1550 nm, the mean CL were 1.54 ± 0.04 dB/optical port (TE) and 1.46 ± 0.1 dB/optical port (TM). For setup 2 (Figure 4a), PDL measurements at 1310 nm yielded mean minimum and maximum losses of 1.50 ± 0.02 dB and 1.67 ± 0.01 dB per optical port, respectively. At 1550 nm, the mean losses ranged from 1.23 ± 0.03 dB to 1.78 ± 0.04 dB per optical port, respectively. The mean coupling losses reported for the PDL measurements are higher than setup 1 because BL, TL, and PL were not subtracted and thus the reported CL included these losses. In addition, the reported CL uncertainties for PDL measurement reflect measurement repeatability and do not include error contributions from bends, tapers, or waveguides. Across the repeated alignments, the measured PDL variations remain small, indicating that minor misalignment or repositioning of the WAFT does not significantly affect PDL. Assessing coupling loss variation due to process variation such as SiN thickness non uniformity, dicing induced facet quality would require measurements across multiple chips. This is beyond the scope of the present work but is planned for the future studies.

Table 3 presents a comparison between our EC results and previously reported state-of-art EC designs. The reported CL values in this work are slightly higher because they include loss contributions from bends, tapers, and waveguide propagation. The intrinsic coupling loss is therefore expected to be lower. The reported losses, thus align well with the existing EC performance in terms of CL. In Table 3, some references report comparable or even lower coupling losses despite using fibers with smaller MFDs. This is because coupling loss depends not only on the MFD but also, on the efficiency of the mode overlap between the fiber and the waveguide. These prior works employed high–numerical–aperture (NA) fibers, which provide stronger mode confinement and therefore enable improved matching to the tightly confined SiN waveguide mode. In addition to using high-NA fibers, these studies also incorporated various coupling-enhancement strategies, such as multisegment tapers for adiabatic mode transformation,^9^ carefully fabricated vertical tapers to expand the optical mode,^11^ multiple taper tips to reduce field discontinuities, and ribbed SiN/silica claddings to increase the vertical mode size and reduce the effective index contrast.^18^ Together, these approaches mitigate scattering at the chip–fiber interface and improve overall edge-coupling efficiency. In contrast, the MFD achievable with the WAFT in our work at the PIC interface remains larger than that obtained using high-NA fibers. Additionally, our design relies on linear tapers, which are simpler to fabricate. In terms of mechanical and packaging considerations, competing edge coupler approaches achieving low CL often rely on V-grooves,^21^ lensed fibers, or 3D-printed tips.^22^ These approaches introduce constraints on mechanical robustness, scalability, and manufacturability, particularly for high-density PICs. By contrast, the glass-based WAFT integrates spot-size converters and fiber-spacing concentrators, enabling flexible on-chip channel pitch down to 15 μm without altering the external fiber pitch. The WAFT supports 1–121 channels, achieving up to 67 channels/mm at the PIC edge, far exceeding conventional EC densities while achieving a competitive CL. Importantly, the WAFT outputs can be prebonded to standard fiber arrays, eliminating the need for active alignment or postbonding pitch transformation. This reduces packaging complexity and mechanical stress compared to conventional fiber-array solutions with a fixed pitch, thereby simplifying assembly and mechanical robustness and compactness. Detailed thermomechanical and packaging considerations for the WAFT–SiN interfaces are provided in Subsection S1e of Section S1 in the Supporting Information. Thus, the key novelty of our approach lies in achieving competitive coupling loss together with practical, scalable packaging that supports very high channel density and flexible pitch adaptation via a glass interposer, without modification of the external fiber array, making it well-suited for WDM and optical-switching systems.

**Table 3:** Comparison of State-of-Art EC Designs

		CL		
λ	Fiber MFD μm	Theo. dB	Exp. dB	port count/mm
1.55 ^8^	10	0.18	0.18 ± 0.12	6–7
1.55 ^9^	4	-	0.37(TE)/0.55(TM)	6–7
1.53–1.57 ^11^	3.2	0.70	1.3	7
1.26–1.36 ^16^	4	1(TE)/1.4(TM)	<1.6(TE)/1.9(TM)	7
1.55 ^15^	10	0.38	0.85(TE)/1.09(TM)	-
1.57 ^18^	6.5 ± 0.5	∼0.6(TE)@ 1550 nm	0.35(TE)/0.9(TM)	5
1.52–1.61 ^25^	2.5	0.58(TE)	1.47(TE)	-
1.52–1.61^a^	10	0.59(TE)@ 1550 nm^b^	W1:1.67–1.86 (TE)	67
		0.97(TM)@ 1550 nm^b^	1.58–1.69(TM)	
			W2:1.35–1.83 @1550 nm	
1.26–1.36^a^	10	0.61(TE)@ 1310 nm^b^	W1:1.55–1.81(TE)	67
		1.35(TM)@ 1310 nm^b^	1.55–1.93(TM)	
			W2:1.42–1.62 @1310 nm	

a This work.

b Simulated WAFT to photonic chip CL.

## 4 Conclusions

Modern photonic interconnects must scale efficiently to meet growing demands for bandwidth and integration density in high-performance PICs. In this work, we demonstrate a compact, low-loss WAFT-assisted fiber-to-SiN edge coupler capable of supporting a high I/O channel density of 67 channels/mm. Fiber-to-chip CL ranges from 1.67–1.86 dB per optical port (TE) and 1.58–1.69 dB per optical port (TM) across the C-band, while in the O-band, losses vary from 1.55–1.81 dB per optical port (TE) and 1.55–1.93 dB per optical port (TM). Polarization-dependent loss (PDL) measurements performed via polarization scanning at 1310 nm yielded CL between 1.42 and 1.62 dB per optical port, and at 1550 nm, between 1.35 and 1.83 dB. By utilizing a glass-based WAFT structure with multiple I/O channels, our design enables broadband, polarization-insensitive coupling without increasing the overall device footprint. This approach offers scalability and compatibility with high-density PICs, providing a versatile solution for modern high-speed photonic systems.

## Supplementary Material



## Data Availability

The data underlying this work are available in ref 26.

## Notes

Supplementary figures; tables; detailed additional data analysis (PDF)
