# Supplementary material for: Glass Interposer Assisted Edge Coupling to SiN Photonic Integrated Circuits
Source: ACS Photonics. 2026 Mar 27;13(8):2120–7. doi: 10.1021/acsphotonics.5c02702 (PMC13088358; doi:10.1021/acsphotonics.5c02702)
Supplement: Supplementary file 1 [file ph5c02702_si_001.pdf]

Supporting Information

Glass interposer assisted edge coupling to SiN photonic integrated circuits

Ipsita Chakraborty,[*,†] Elliot Sandell,[†] Thalía Domínguez Bucio,[†] Glenn Churchill,[†] Xingshi Yu,[†] Michail Symeonidis,[‡] Hawraa Atwi,[¶] Clement Fresse,[¶] James Gates,[†] and Frederic Gardes[†]

†*Optoelectronics Research Centre, University of Southampton, United Kingdom, SO17 1BJ*

‡*Fraunhofer Institute for Reliability and Microintegration IZM, Gustav-Meyer-Allee 25, 13355 Berlin, Germany*

¶*TEEM Photonics, 61 chemin du Vieux Chêne - 38240 Meylan Cedex - France*

E-mail: ic1m23@soton.ac.uk

Number of pages: 14

Number of figures: 6

Number of tables: 3

S1 Additional Data or Analysis

S1a Fabry–Perot (FP) Analysis of Broadband Coupling-Loss Oscillations

To quantitatively analyze the experimentally observed spectral oscillations in the measured coupling-loss spectra shown in Fig. 3(c) (in manuscript), we applied a Fabry–Perot interference model. A fast Fourier transform (FFT) was performed on the measured broadband coupling-loss spectra in both the O band and C band for transverse-electric (TE) and transverse-magnetic (TM) polarizations. The FFT magnitude, plotted as a function of optical path difference (OPD) is shown in Fig. A, reveals distinct cavity lengths responsible for the observed spectral oscillations.

From the extracted OPD peaks, the corresponding fringe spacing was estimated using

$$\Delta\lambda \approx \frac{\lambda_0^2}{\mathrm{OPD}}, \tag{S1}$$

where $\lambda_0 = 1310$ nm for the O band and $\lambda_0 = 1550$ nm for the C band. The effective cavity length was then calculated using the Fabry–Perot free spectral range relation

$$L \approx \frac{\lambda_0^2}{2n_g\Delta\lambda}, \tag{S2}$$

where n_g is the group index of the SiN waveguide. The group indices for straight, taper, and bend sections were obtained from Ansys Lumerical FDE mode simulations, as summarized in Table S1 of SI.

The physical length of the SiN test structure is approximately

$$L_{\mathrm{phys}} = 2L_{\mathrm{straight,min}} + 2\left(L_{\mathrm{taper}} + L_{\mathrm{straight}}\right) + \pi R_{\mathrm{bend}}, \tag{S3}$$

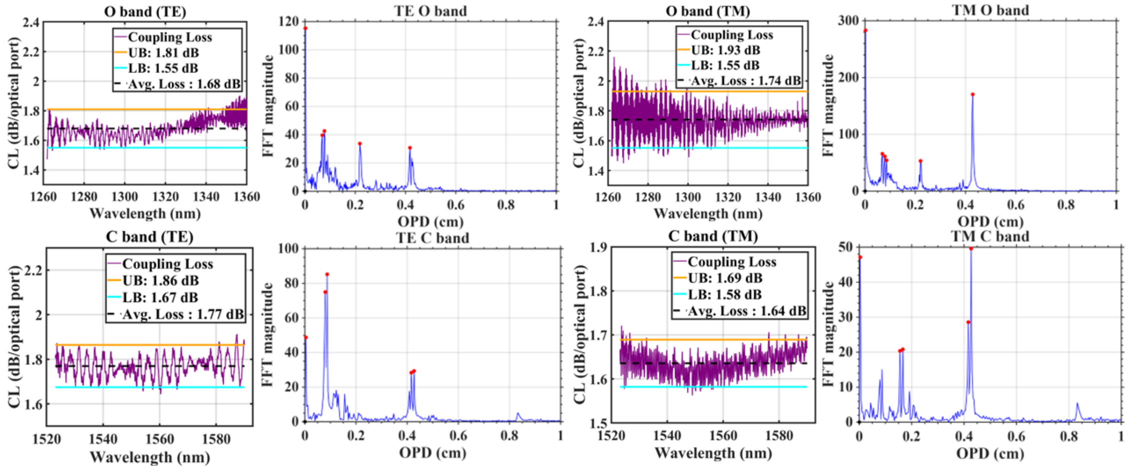

Figure S1: Broadband coupling-loss evaluation across the O band and C band for TE and TM polarizations. The taper length is $L = 200\ \mu$m for all cases, with taper tip width $t = 230$ nm at 1310 nm and $t = 290$ nm at 1550 nm. FFT magnitude is plotted as a function of optical path difference (OPD) for both O and C bands and both for both TE and TM polarizations. The FFT is performed on the broadband coupling-loss spectra shown in Fig. 3(c) of the main manuscript.

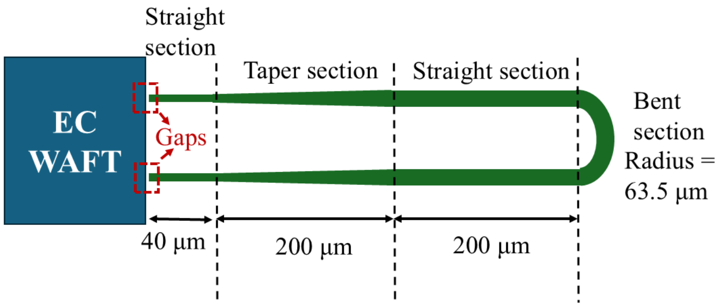

Figure S2: SiN test structure used in the cutback method.

which evaluates to

$$L_{\text{phys}} = 2 \times 40 + 2 \times (200 + 200) + \pi \times 63.5 \approx 1080\ \mu\text{m}. \tag{S4}$$

The SiN test structure consists of multiple sections, including taper, straight, and bend regions. Therefore, the final group index $n_{g,\text{final}}$ represents an effective group index that accounts for the varying group indices of each section. This effective group index is calculated

Table S1: Group index calculation for different sections of the SiN test structure. Values marked with * are obtained from Ansys Lumerical FDE simulations.

Config.	$n_{g,\text{straight}} = n^*_{g,\text{taper,max}}$	$n^*_{g,\text{taper,min}}$	$n_{g,\text{taper}}$	$n^*_{g,\text{bend}}$	$n_{g,\text{final}} \pm$ error
TE 1310 nm	2.0538	1.5449	1.7994	1.6448	1.8461 ± 0.176
TM 1310 nm	2.0028	1.6483	1.8255	2.0025	1.9093 ± 0.1105
TE 1550 nm	2.0285	1.5420	1.7853	1.6411	1.8307 ± 0.1676
TM 1550 nm	1.9431	1.6266	1.7848	1.9473	1.8618 ± 0.1005

using a length-weighted average:

$$n_{g,\text{final}} = \frac{\sum_i L_i n_{g,i}}{\sum_i L_i}, \tag{S5}$$

where L_i and $n_{g,i}$ are the length and group index of the ith section, respectively. The associated error is estimated as

$$n_{g,\text{final error}} = \frac{\sum_i L_i \left(n_{g,i} - n_{g,\text{final}}\right)^2}{\sum_i L_i}. \tag{S6}$$

The group index of the taper section is calculated as

$$n_{g,\text{taper}} = \frac{n_{g,\text{taper,min}} + n_{g,\text{taper,max}}}{2}. \tag{S7}$$

Using group indices obtained from Ansys Lumerical FDE mode simulations of the straight, taper, and bend sections, we identify multiple Fabry–Perot cavities contributing to the observed ripples in the coupling-loss spectra. A short cavity corresponding to an effective length of approximately 10–20 μm with $n_g = 1$ is consistently observed across all polarizations and wavelength bands, which is attributed to the finite air gap between the WAFT and the SiN waveguide facet. Additional peaks correspond to cavity lengths associated with reflections from the bend section and the input/output tapers (approximately 200 μm). Furthermore, cavity lengths of approximately 1.1–1.2 mm are extracted, in close agreement with the physical length of the SiN test structure, confirming Fabry–Perot behavior arising from reflections at the input and output facets. Minor discrepancies in the extracted cavity lengths arise from

Table S2: Cavity length L_c calculation based on FFT of the broadband spectrum using corresponding group indices.

Config.	OPD (cm)	$\Delta\lambda$ (nm)	n_g	L_c (μm)	Cause
TE O band	0.002143	80.083	$n_{g,\text{air}} = 1$	10.71	Air gap (WAFT-SiN facet)
	0.077144	2.225	$n_{g,\text{taper}} = 1.799$	214.30	Input/output taper section
	0.068573	2.503	$n_{g,\text{bend}} = 1.644$	208.41	Reflections involving bend section
	0.218575	0.785	$n_{g,\text{final}} = 1.846$	592.07	Taper–bend FP cavity
	0.417864	0.411	$n_{g,\text{final}} = 1.846$	1130.85	Full SiN test structure FP cavity
TM O band	0.002143	80.083	$n_{g,\text{air}} = 1$	10.71	Air gap (WAFT-SiN facet)
	0.428578	0.400	$n_{g,\text{final}} = 1.909$	1123.50	Full SiN test structure FP cavity
	0.068573	2.503	$n_{g,\text{taper}} = 1.825$	187.78	Input/output taper section
	0.079287	2.164	$n_{g,\text{bend}} = 2.000$	198.25	Reflections involving bend section
	0.085716	2.002	$n_{g,\text{taper}} = 1.825$	234.78	Input/output taper section
	0.220718	0.778	$n_{g,\text{final}} = 1.909$	577.00	Taper–bend FP cavity
TE C band	0.086839	2.767	$n_{g,\text{taper}} = 1.785$	243.17	Input/output taper section
	0.079603	3.018	$n_{g,\text{taper}} = 1.785$	222.95	Input/output taper section
	0.003618	66.399	$n_{g,\text{air}} = 1$	18.09	Air gap (WAFT-SiN facet)
	0.426960	0.563	$n_{g,\text{final}} = 1.830$	1165.51	Full SiN test structure FP cavity
	0.416105	0.577	$n_{g,\text{final}} = 1.830$	1137.24	Full SiN test structure FP cavity
TM C band	0.426960	0.563	$n_{g,\text{final}} = 1.861$	1146.01	Full SiN test structure FP cavity
	0.003618	66.399	$n_{g,\text{air}} = 1$	18.09	Air gap (WAFT-SiN facet)
	0.416105	0.577	$n_{g,\text{final}} = 1.861$	1118.21	Full SiN test structure FP cavity
	0.166442	1.443	$n_{g,\text{final}} = 1.861$	447.13	Taper–straight section cavity
	0.155587	1.544	$n_{g,\text{final}} = 1.861$	417.88	Taper–straight section cavity

the use of an effective group index weighted across different waveguide sections. The cut-back SiN test structure consists of two nominally identical arms; however, small variations in physical dimensions introduced during dicing can lead to differences in effective optical path length, resulting in multiple closely spaced cavity peaks in the FFT spectrum. This Fabry–Perot analysis quantitatively explains air-gap-induced Fresnel reflections and cavity formation within the SiN waveguide sections, thereby bridging the discrepancy between idealized simulations and measured coupling-loss spectra. These oscillations, arising from both air-gap-induced Fresnel reflections and internal reflections at transitions between waveguide components (e.g., taper-to-straight and straight-to-bend sections), could be mitigated by

applying an index-matching liquid or adhesive at the air–waveguide interface, optimizing waveguide transitions, and employing Euler bends instead of constant-radius bends to further reduce reflections within the SiN cavity.

S1b Polarization Mode Dispersion (PMD) in the WAFT

The WAFT is a glass-based ion-diffused waveguide, which is intrinsically low-birefringence and not expected to introduce measurable polarization mode dispersion (PMD) over the short device length ($\approx$ 7 mm). The waveguide geometry is symmetric and relies on smooth adiabatic transitions (spot-size conversion, depth transition, and pitch adaptation), making the platform polarization-insensitive by design. TEEM specifications report low polarization extinction ratio ($\leq$ –55 dB) and low crosstalk ($\leq$ –40 dB), and the low PDL ($\leq$ 1 dB) measured experimentally further supports the absence of significant polarization-related dispersion effects. PMD was therefore not explicitly modeled or measured, as it is expected to be negligible for high-speed data transmission in this configuration.

S1c PDL behavior across the O and C bands

We have also studied broadband PDL behavior of the WAFT across the O and C bands as shown in Fig. S3 of SI. In both bands, the PDL remains low overall, varying smoothly with wavelength and showing no monotonic increase or decrease across the band. In the O band, the PDL stays below 1.05 dB and exhibits only weak wavelength dependence, despite local peaks and dips observed in the absolute insertion loss. In the C band, localized PDL variations are observed, with two maxima near 1530 nm and 1570 nm reaching about 1 dB. These variations are bounded and do not indicate strong or broadband polarization sensitivity. Overall, the measured behavior demonstrates stable polarization performance over both wavelength bands relevant to practical operation.

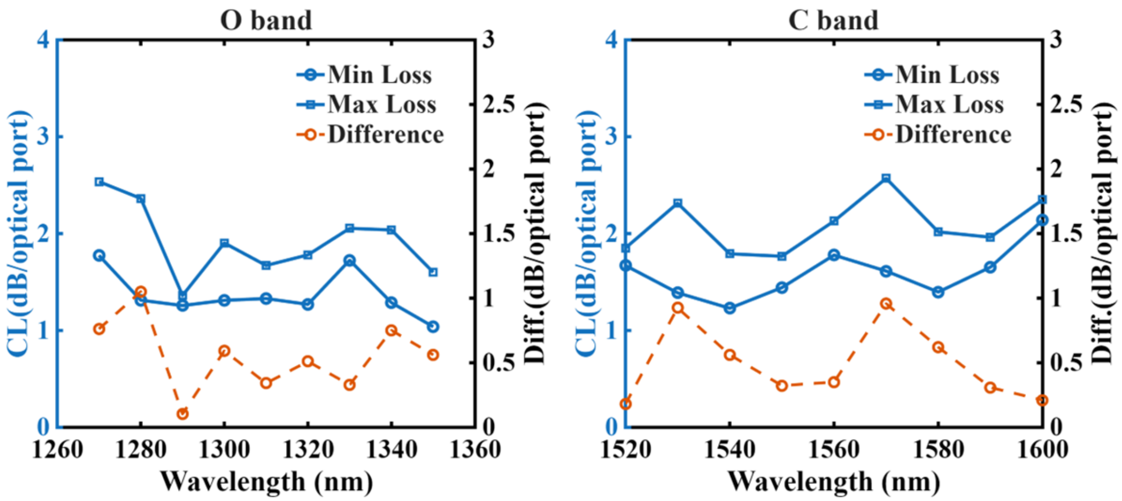

Figure S3: PDL variation across the O and C bands for t = 230 nm (optimized near 1310 nm) and t = 290 nm (optimized near 1550 nm) with L = 200 µm.

S1d Wavelength-Dependent Behavior of Bend loss (BL), Taper loss (TL), and Propagation Loss (PL)

Fig. S4 of SI shows the wavelength dependent behavior of TL, BL and PL. The observed wavelength dependence arises primarily from changes in effective refractive index and mode field distribution away from the design wavelength, which affect mode confinement, bend radiation, and taper adiabaticity. In the O band (1260–1360 nm), TL for both TE and TM polarizations remains approximately in the range of 0.2–0.3 dB per taper, with a slight increase near the long-wavelength edge around 1340 nm. In the C band (1520–1600 nm), TL exhibits weaker wavelength dependence, varying by less than 0.06 dB across the band for both polarizations. BL shows stronger wavelength dependence in the O band, particularly for TM polarization, decreasing monotonically from approximately 0.22 dB/bend at shorter wavelengths to nearly zero at longer wavelengths. The TE-mode BL follows a similar trend, decreasing from about 0.16 dB/bend to below 0.07 dB/bend. In contrast, BL in the C band remains low ($<$ 0.12 dB/bend) for both polarizations and varies weakly with wavelength. Regarding the impact of propagation loss (PL) on broadband CL, the maximum SiN waveguide

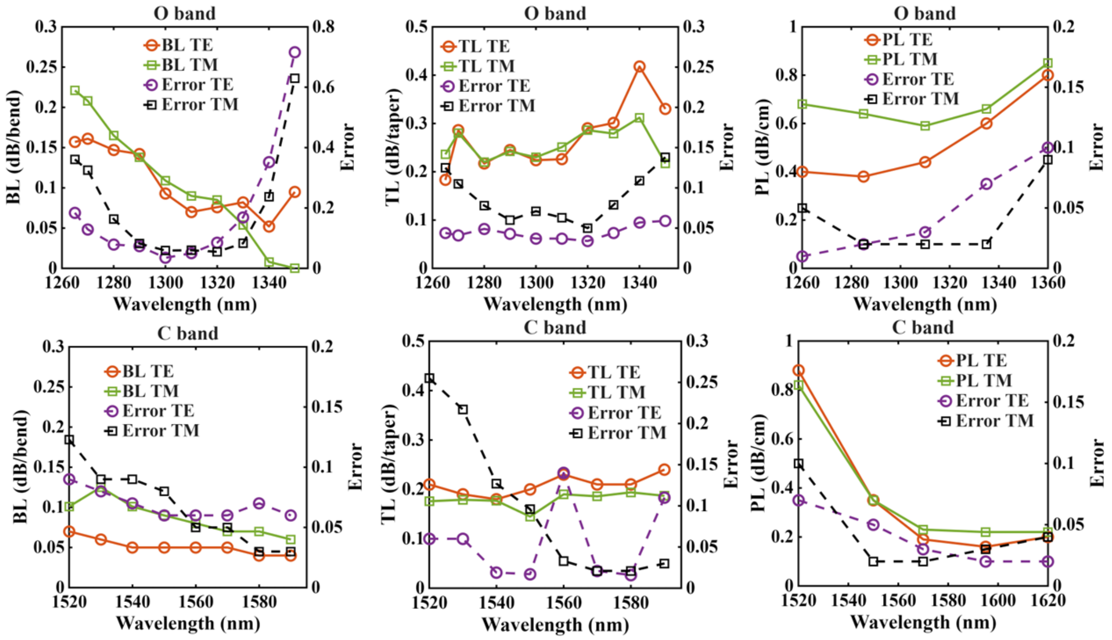

Figure S4: Broadband variation of BL, TL, and PL for TE and TM polarizations across the O and C bands. TL was measured for t = 230nm (optimized at 1310 nm) and t = 290nm (optimized at 1550 nm).

length in our devices is 1080 µm. Since PL is reported in dB/cm, the total variation across the O and C bands is less than 0.1 dB, which has a negligible effect on the broadband CL. Although BL, TL, and PL vary with wavelength, the broadband CL measurements (Fig. 3(c) in manuscript) inherently includes the wavelength-dependent contributions of these losses, since Bl, TL and PL were not subtracted from the total CL. This leads to a slightly higher CL and modest spectral variation, particularly near the band edges. Thus, broadband CL measurements reflect the true operational behavior of the device over the full wavelength range of interest.

S1e Thermomechanical and Packaging Considerations for WAFT–SiN Interfaces

The materials and assembly approach are compatible with established photonic packaging practices.

- **Thermomechanical compatibility:** The WAFT uses an ion-exchanged glass inter-

poser whose coefficient of thermal expansion (CTE) can be adjusted to match that of Si / SiO2. This yields a moderate CTE mismatch at the WAFT–chip interface that can be managed with packaging choices. Similar glass-interposer based photonic substrates have been shown to be compatible with standard thermal cycling and board-level assembly processes[1,2](references in SI).

- **Adhesive/bonding concept:** In an industrial implementation, the WAFT would typically be attached to a thermally matched carrier and butt-coupled to the PIC edge using either (i) a thin bead of low-shrinkage, low-modulus epoxy at the outer perimeter of the WAFT–chip interface, or (ii) a mechanical clamp with underfill away from the optical aperture. Such epoxies are widely used for fiber-array and microlens attachment in telecom modules and are qualified under industrial standards (e.g. Generic Reliability Assurance Requirements for Passive Optical Components) for thermal cycling, damp heat, mechanical shock and vibration. By keeping the adhesive layer at the optical interface to a few micrometers and using compliant formulations, the induced stress on the buried SiN waveguides remains small.

- **Stress-reduction approaches:** The SiN waveguides are embedded in a thick oxide stack, and the WAFT waveguides are buried into the glass, leading to a tight confinement of the guided mode, securing it against stress-induced perturbations. If needed, the waveguides could be designed to be offset from the coupling facet further reducing the impact of stress at the interface. WAFT-based SSC/interposer products are already deployed in commercial PIC packaging, underscoring industrial relevance[1–3](references in SI).

S1f Index Matching Materials and Long-Term Reliability

- **Index matching liquids vs. cured adhesives:** The index matching liquids we mention are primarily intended as a laboratory tool to suppress Fresnel fringes during

characterization. Such liquids are generally not suitable for long term telecom/datacom packaging because of evaporation, migration and outgassing. For an industrial module, the interface would instead be filled with a thin layer of a cured index matching adhesive (typically n $\approx$ 1.44–1.48) or treated with an anti-reflection (AR) coating on one or both facets.

- **Compatibility with long term operation:** UV or thermally curable index matching epoxies designed for photonic packaging have been widely used for decades in fiber pig tailing, fiber array attachment and lens assemblies. When industry graded formulations are used, they meet the relevant standards requirements (e.g. GR 1221/GR 468) for thermal cycling, high temperature storage and damp heat, with insertion loss drifts that are acceptable for telecom/datacom systems.[4,5] For the WAFT–SiN interface, the required adhesive thickness is only a few micrometers, so the added optical path is short, and the thermos-mechanical stress transmitted to the underlying SiN waveguides is minimal.

- **Impact on mechanical robustness and outgassing:** A cured index matching adhesive generally improves mechanical robustness, because it immobilizes the WAFT relative to the chip and increases the bonded area compared with a bare butt contact. To minimize stress and potential chip bow, low modulus (or at least moderate modulus) formulations with low shrinkage should be selected. Regarding outgassing and contamination, adhesive systems qualified under industrial standards are specifically tested for volatile content and for the absence of optically detrimental deposits on nearby surfaces. Using such materials, we do not expect the index matching adhesive to be the limiting factor for meeting telecom/datacenter environmental specifications.

S1g Design Considerations for Bend Radius

A bend radius of 63.5 µm was chosen for the cutback test structure to match the 127 µm pitch of the WAFT channels and to closely approximate the 60 µm radius identified through simulation (see Fig. S5 of SI) as providing the optimal trade-off between bend loss and overall propagation loss for the SiO_2/SiN/SiO_2/Si platform. However, these constant-radius bends may introduce cavity effects within the SiN waveguide, as evident by distinct peaks in the FFT analysis of the broadband transmission spectrum. Implementing Euler bends offers a practical alternative to constant radius bend by reducing reflections and enabling smooth, low-loss transitions between straight and bend waveguide sections

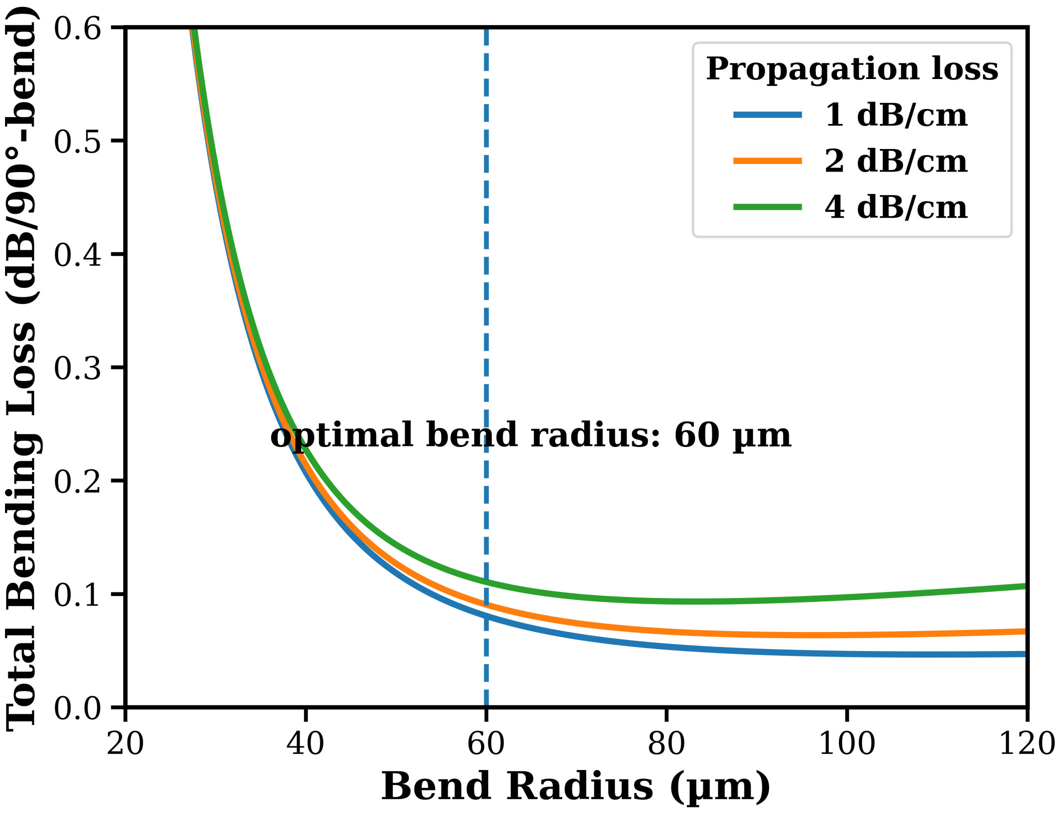

Figure S5: Simulated bending loss (BL) as a function of bend radius for different assumed propagation losses at 1550 nm wavelength. Our fabricated SiN chip exhibits propagation losses of 0.35 dB/cm at 1550 nm.

S2 Supplementary Figures

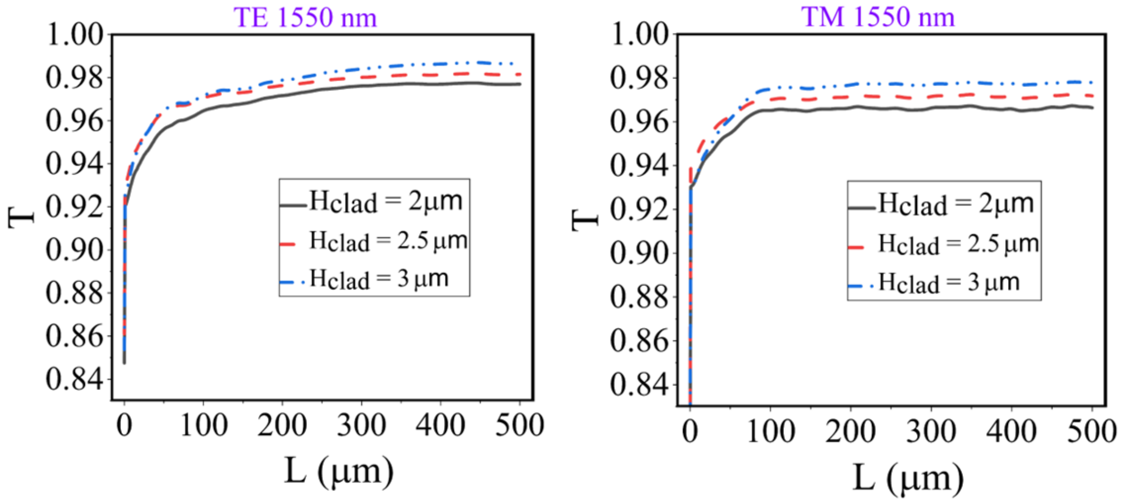

Figure S6: Transmission versus taper length for different cladding thicknesses at 1550 nm, shown for TE and TM polarizations.

S3 Supplementary Tables

The EC-WAFT is based on a buried ion-diffused glass waveguide that transitions adiabatically to a surface waveguide at the coupling interface with the SiN chip. This surface section is the part interacting optically with the PIC. The glass refractive index is wavelength-dependent, and the ion-diffusion process creates a smooth, continuous refractive-index increase near the surface, described by a lateral and vertical diffusion profile. This results in a near-Gaussian optical mode. Table S3 of SI shows the properties of internal WAFT section consisting of buried ion–diffused waveguide.

Table S3: Buried ion-diffused waveguide parameters (internal WAFT section)

Category	Properties	Values
**Buried ion-diffused waveguide** (internal WAFT section)	Wavelength-dependent glass substrate refractive index	$n \approx 1.509$–1.510 @ 1550 nm<br>$n \approx 1.524$–1.525 @ 1310 nm
	Maximum refractive index increase due to diffusion	$\Delta n \approx 0.05$
	Effective diffusion depth (vertical, Gaussian-like)	$\sigma \approx 1.45\ \mu$m
	Typical buried waveguide effective mode size (fiber side)	MFD $\approx$ 10 μm (matched to SMF via spot-size converters)
**Surface waveguide** (EC-WAFT section interacting with SiN PIC)	Mask opening width W	$\sim 2.0\ \mu$m
	Peak refractive index in surface region	$n \approx 1.518$–1.525 (depending on wavelength and polarization)
	Mode-field diameter at PIC interface	Vertical MFD: ~ 2.7–$3.2\ \mu$m<br>Horizontal MFD: ~ 3.7–$4.3\ \mu$m<br>(TE/TM, 1310 nm & 1550 nm — see Table 1 in manuscript)
	Refractive-index profile from TEEM	Near-Gaussian mode at PIC interface providing intrinsic tolerance to small misalignments
	Pitch tolerance	$\pm 0.05\,\mu$m (non-cumulative tolerance between channels, e.g., channel X and Y)

References

(1) Brusberg, L.; Zakharian, A. R.; Kocabaş, Ş. E.; Yeary, L. W.; Grenier, J. R.; Terwilliger, C. C.; Bellman, R. A. Glass substrate with integrated waveguides for surface mount photonic packaging. Journal of Lightwave Technology **2020**, 39, 912–919.

(2) Peng, B.; Barwicz, T.; Sahin, A.; Houghton, T.; Hedrick, B.; Bian, Y.; Rakowski, M.;

Hu, S.; Ayala, J.; Meagher, C.; others A CMOS compatible monolithic fiber attach solution with reliable performance and self-alignment. Optical Fiber Communication Conference. 2020; pp Th3I–4.

(3) Vanmol, K.; Saurav, K.; Panapakkam, V.; Thienpont, H.; Vermeulen, N.; Watté, J.; Van Erps, J. Mode-field matching down-tapers on single-mode optical fibers for edge coupling towards generic photonic integrated circuit platforms. Journal of lightwave technology **2020**, 38, 4834–4842.

(4) Telcordia Technologies Generic Reliability Assurance Requirements for Passive Optical Components. GR-1221-CORE, 2000.

(5) Telcordia Technologies Generic Reliability Assurance Requirements for Optoelectronic Devices Used in Telecommunications Equipment. GR-468-CORE, 2000.
